# Infection prevention and early warning in neonatal intensive care unit based on physiological sensor monitoring

**DOI:** 10.3389/fbioe.2023.1241287

**Published:** 2023-08-30

**Authors:** Chao Tang, Fenfang Lei, Jirong Liu, Fengxiang Gong

**Affiliations:** School of Nursing, Shao Yang University, Shaoyang, China

**Keywords:** early warning, ward infection, neonatal intensive care unit, physiological sensors, wireless physiological sensor network

## Abstract

The infection rate in the Neonatal Intensive Care Unit (NICU) is very high, which is also one of the important causes of morbidity and even death in critically ill neonates and premature infants. At present, the monitoring system of the Neonatal Intensive Care Unit is not very complete, and it is difficult to provide early warning of neonatal illness. Coupled with the untimely response measures, it has brought certain difficulties to the ward’s infection prevention and control work. The rapid development of the Internet of Things (IoT) in recent years has made the application fields of various sensor devices more and more extensive. This paper studied infection prevention and early warning in the Neonatal Intensive Care Unit based on physiological sensors. Combined with a wireless network and physiological sensors, this paper built an intelligent monitoring system for the Neonatal Intensive Care Unit, which aimed to monitor various physiological data of newborns in real-time and dynamically, and gave early warning signals, so that medical staff could take preventive measures in time. The experiments showed that the monitoring system proposed in this paper could obtain the physiological information of neonates in time, which brought convenience to prevention and early warning work, and reduced the infection rate of neonatal wards by 7.39%.

## 1 Introduction

The emergence of the Neonatal Intensive Care Unit has given many neonatal critically ill patients a chance for treatment and survival. With the improvement of medical levels and the rapid development of the economy, many hospitals have established Neonatal Intensive Care Units, so that many critically ill neonatal patients can be treated. Typically, most newborns require ventilator-assisted ventilation, and their immune functions are immature and susceptible to infection, which poses a challenge to the NICU. The monitoring system in the ward is no longer able to satisfy the needs of the current Neonatal Intensive Care Unit due to a lack of technical improvements, which also makes relevant infection interventions challenging. An attempt and innovation is the use of physiological sensors to detect infections early in the Neonatal Intensive Care Unit.

Ward infection is one of the important factors affecting the health of patients. At present, many scholars have also joined the ranks of research onward infection. Hegazy A A investigated the infection in the Intensive Care Unit (ICU) and found that the infection rate was higher in the wards involving the respiratory and genitourinary systems than in other wards (Hegazy et al., 2019). Behnke M proposed a ward liaison monitoring method, which had high-efficiency inward data collection and could provide certain data support for the prevention of hospital ward infection (Behnke et al., 2017). Muloi D conducted a study of hospital ward infections in geriatric patients and found that the reduction in non-essential ward transfers in elderly patients would reduce the incidence of infection in this population (Muloi et al., 2020). Kelly M S evaluated the application effect of the “3 + 1”model in the prevention of Corona Virus Disease 2019 (COVID-19) (new coronary pneumonia) ward infection. Among them, “3”represented the epidemic prevention and control system, personnel management and prevention and control measures, and “1”represented COVID-19 detection. Finally, it was found that this model had a good effect in preventing ward infection (Kelly et al., 2019). Dicky discussed the impact of a ventilation system on the ward and concluded that poor ventilation was the main factor leading to the high cross-infection rate in the ward (Dicky et al., 2019). Deng pointed out that the high infection rate in the ward of tracheotomy patients after craniocerebral injury might be related to various factors. To reduce the infection rate, antibiotics could be used rationally, an aseptic operation could be strengthened, as well as the immunity of patients could be improved (Deng et al., 2017). Hazelton M S pointed out that ward cleaning, disinfection and isolation were important links in the prevention and control of ward infection. The strengthening of this work was conducive to the control of ward infection (Hazelton et al., 2017). These studies onward infections are relatively specific, but they do not involve physiological sensor monitoring.

A physiological sensor is a kind of intelligent sensor device, which is currently used in medicine, healthcare, monitoring and other fields. Hong Y integrated the wireless communication network into the physiological sensor and finally realized the remote acquisition of the patient’s physiological signal, which freed the patient from the shackles of medical monitoring equipment (Hong et al., 2019). Chen C developed a smart physiological sensor using state-of-the-art optical chip technology. The tests showed that the sensor had a good effect in obtaining the patient’s electrocardiogram (ECG) (Chen et al., 2021). Lin X designed a wireless sensor network monitoring system combined with physiological sensors. The simulation results showed that the sensor could transmit data to network nodes in real time with high reliability (Lin et al., 2020). Liu J designed a paper-based pressure physiological sensor, which showed high performance in monitoring the user’s finger movement, vocal cord vibration, and wrist pulse (Liu et al., 2017). Srinivasa M G applied the physiological sensor to the wearable physiological monitoring system and discussed the common problems of the wearable physiological monitoring system, as well as gave specific solutions (Srinivasa and Pandian, 2019). Chen T S developed a light-gathering structure with an elliptical reflector and used it for multi-parameter physiological sensor monitoring, and finally, the monitoring efficiency and reliability of the sensor were improved (Chen et al., 2019). Williams D used physiological sensors to form a comprehensive body-worn monitoring system, which achieved good results in real-time monitoring of subjects’ physiological and psychological conditions (Williams and Jeremiah, 2020). The above studies on physiological sensor monitoring are relatively detailed, but they have not been applied to infection prevention and control in the Neonatal Intensive Care Unit.

Neonatal Intensive Care Unit are characterized by high infection rates and difficult monitoring. This article developed a monitoring system using physiological sensors in accordance with the peculiarities of the Neonatal Intensive Care Unit. The wireless network was set up and the system’s blood pressure, pulse, breathing, and other modules matched to enable the real-time transfer of neonatal physiological data. In this study, the wireless physiological sensor network was analyzed and calculated using the sensor network timing synchronization protocol in order to maintain data synchronization with the network at all times. In addition, this paper combined monitoring and early warning methods to give infection prevention measures in Neonatal Intensive Care Unit.

## 2 Physiological sensors and node distribution

Physiological sensors are an important perception and component of the Internet of Things, which is also a sensing device used in healthcare, disease monitoring, and prevention (Diwakar et al., 2022). The working principle of physiological sensors is to be placed or implanted inside the human body to obtain human physiological information data (Talaat and El-Balka, 2023).

**FIGURE 1:**
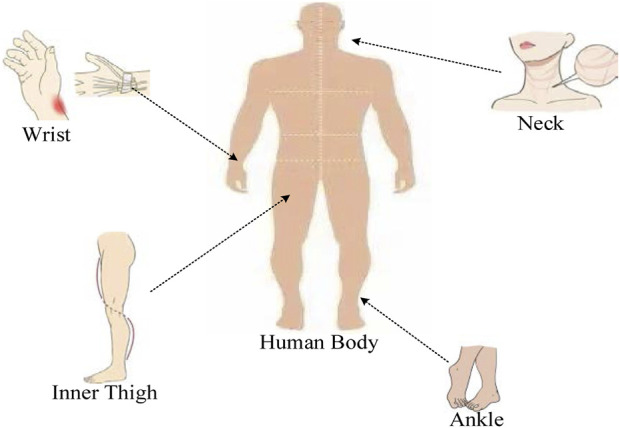
Pulse monitoring site based on physiological sensors.

Physiological sensor nodes include sensing nodes and central nodes, which are mainly placed on the human body in a wearable form (Golgouneh and Tarvirdizadeh, 2020). The sensing node would first collect the physiological information of the human body, and then transmit the data to the central node. The sensing node is usually placed on a specific part of the body (such as the chest, wrist, and earlobe (Yamada, 2018). As shown in Figure 1, each type of physiological information may have different monitoring sites. For example, the monitoring sites for pulse may be wrists, ankles, necks, inner thighs, and so on. The central node can further analyze, process and fuse the data sent by the sensing node. Through the wireless or the Internet, the data is transmitted to the patient, hospital, patient family or other storage terminal. In order not to affect the daily activities of the human body, the central node is generally placed on the waist of the human body.

## 3 Physiological sensor-based infection prevention and monitoring method in neonatal intensive care unit

(1) Overall architecture of the ward monitoring system

**FIGURE 2:**
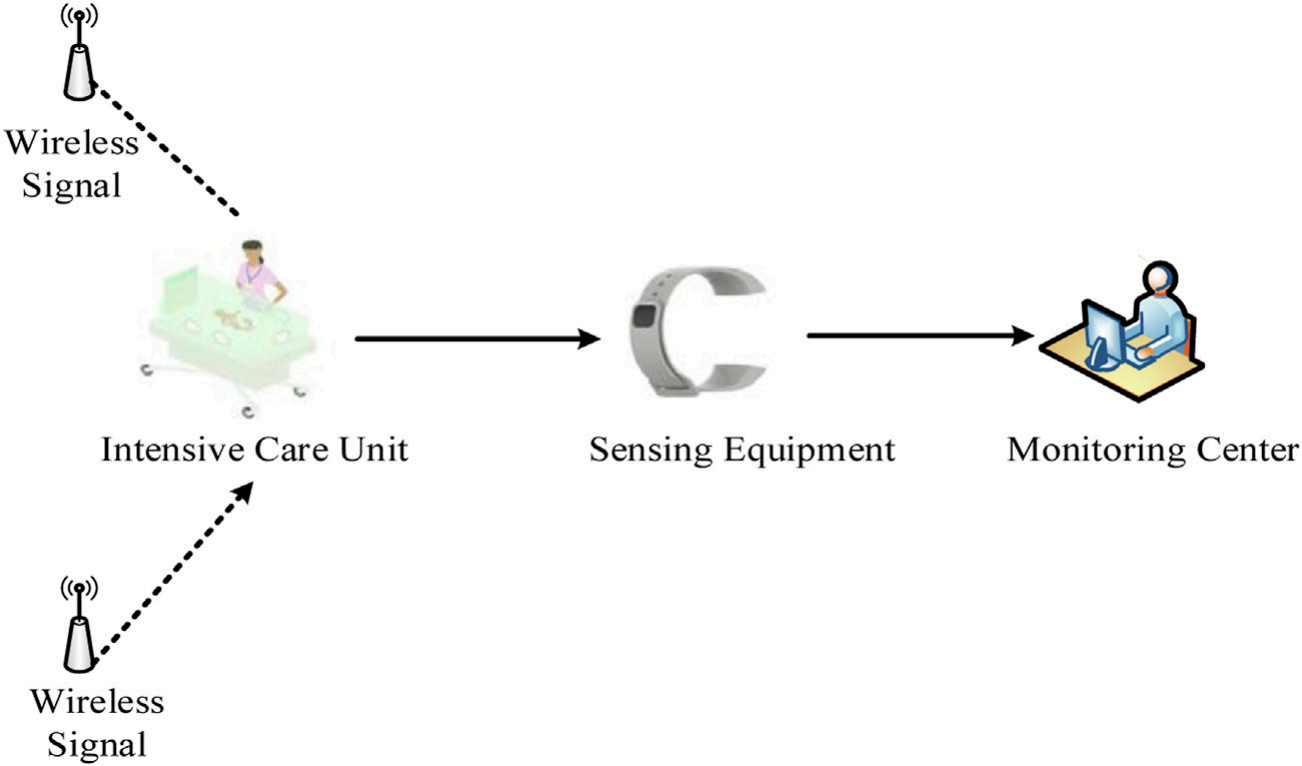
Overall architecture of the monitoring system in the Intensive Care Unit.

Figure 2 shows the overall architecture of the monitoring system in the Neonatal Intensive Care Unit, which can be divided into three parts: the Intensive Care Unit, the sensing equipment, and the monitoring center. In the Intensive Care Unit, several wireless signal transmitters would be placed for the work of the sensing equipment. The baby’s wrist or ankle would be fitted with sensing technology that can continuously monitor the baby’s temperature, pulse, and blood pressure. Additionally, it has an alarm function, which will sound when a newborn’s physiological data is abnormal and will be promptly transmitted to the monitoring centre so that the centre’s medical staff can formulate a response strategy for the particular circumstance.

(2) System modules and functions

**FIGURE 3:**
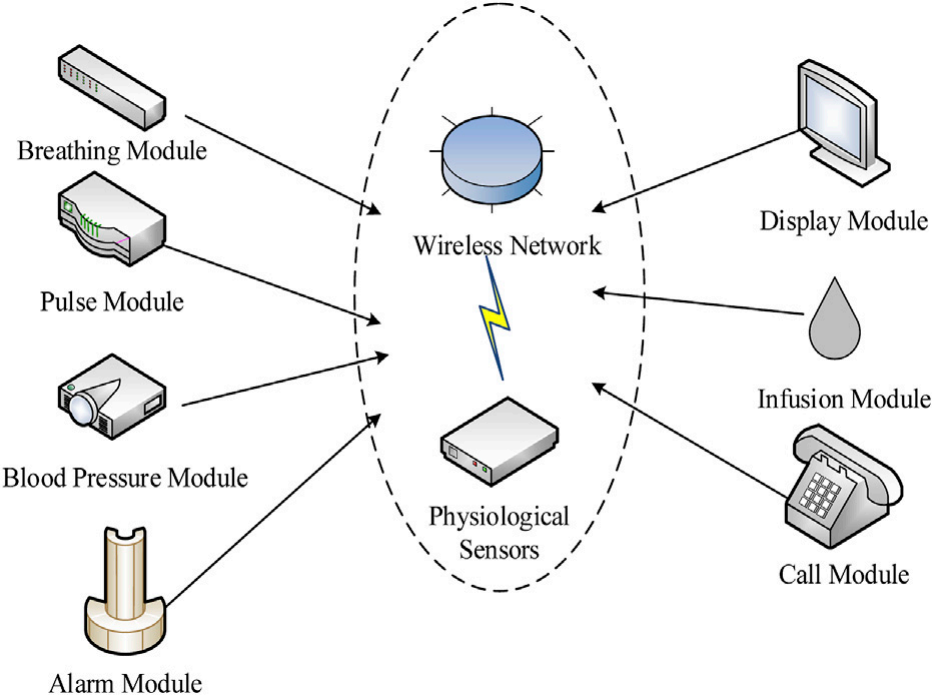
The specific modules and system functions of the monitoring system.

As shown in Figure 3, the system modules include the blood pressure module, the pulse module, the infusion module, the breathing module, the alarm module and the display module. Among them, the blood pressure, pulse and display modules are system hardware. The blood pressure measurement module uses a pressure physiological sensor to directly convert the blood pressure signal of the arterial blood to the blood vessel wall into an electrical signal. After being amplified and regulated, the electrical signal is sent to the conversion module to be converted into a digital signal. After the numerical conversion adjustment and display of the single-chip system, it is used as the detection data of blood pressure. The pulse module tests the heart rate waveform and pulse, and adopts the reflective photoelectric sensor measurement method, so as to integrate a special sensor for detection and obtains the fingertip heart rate data through the finger clip. The sampling principle of the sensor is to emit invisible light through a high-penetration infrared emitting tube to irradiate the fingertip. Opposite the infrared transmitting tube is the corresponding infrared receiving tube. At different times of the heartbeat, the blood volume of the fingertip capillaries is different. The infrared light of the transmitting tube passes through the finger, and the infrared receiving tube receives the amplified signal to judge the heart rate according to the infrared light passing through the finger. The display module mainly displays heart rate waveform, blood pressure, infusion rate, breathing state, neonatal information, etc.

The main functions of the monitoring system are.

a Monitoring and collection of heart rate sensor signals: The output of heart rate signal and heart rate waveform is completed by infrared emission controlled by the heart rate module. Specifically, the single-chip microcomputer is used to collect signals, and the heart rate value and the waveform are presented on the display through the operational amplifier comparison circuit in the unit. Once the heart rate exceeds the standard value or falls below the standard value, the alarm signal would be issued, so that the medical staff can know the emergency of the newborn at the first time.
b Monitoring and collection of breathing signals: The thermal resistance sensor is used and a thermal device is placed under the nose or mouth of the newborn to determine the temperature of the breathing gas of the newborn. Thermal devices are small and easy to use. During the monitoring and identification process, there would be no uncomfortable feeling, and sleep and normal rest would not be affected. After the effective sampling signal is extracted by the signal filter circuit, the respiratory analog signal is converted into a digital signal by the processor, and the counting and display functions are realized at the same time. If a pause in the breathing signal is detected for more than 10 seconds, the alarm would sound an alarm signal
c Blood pressure signal monitoring and collection: By using a pressure sensor, the sensor membrane generates strain under the influence of external pressure and transmits it to the strain gauge. After the strain gauge detects the disturbance, the resistance value of the four arms of the bridge changes, and the signal measurement circuit converts it into an external power supply analogue quantity, as well as the blood pressure value is finally displayed on the display through analogue-to-digital conversion.
d Signals from infrared photoelectric infusion sensors are being monitored and collected: The speed of the droplets above the dripper is measured using infrared sensor detecting technology. The infrared sensor transforms the light into a current signal for emission after the infrared transmitter emits infrared light, which then travels via the dropper to illuminate the receiving phototransistor. The light is strong when there are no droplets in the dropper, and the sensor produces a high-quality signal; the light is dim when a droplet enters the dropper, and the sensor produces a low-quality signal. The microcontroller would be used to process the sensor signal that was received and compute the infusion rate.

(3) Prevention and control measures

**FIGURE 4:**
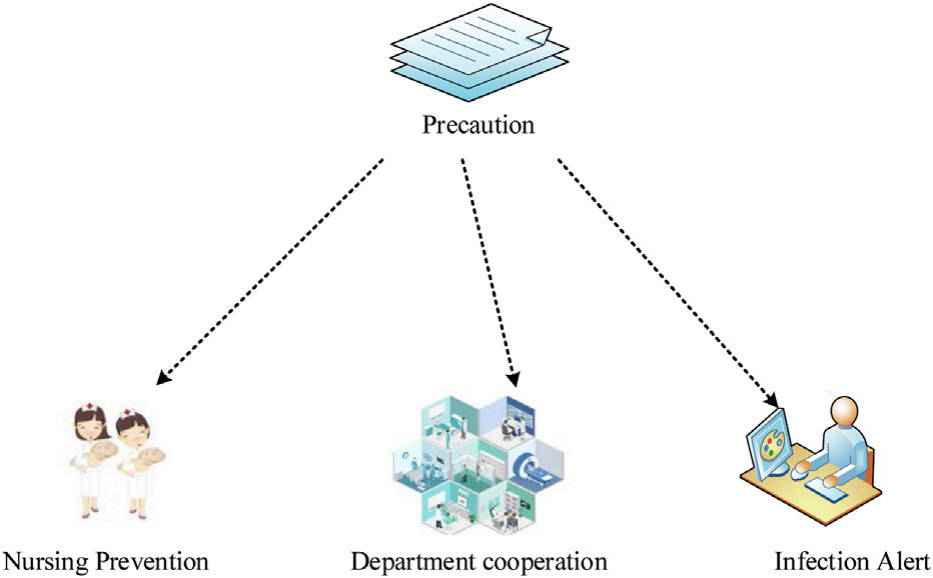
Infection Prevention and control measures in the Neonatal Intensive Care Unit.

As shown in Figure 4, the infection prevention and control measures in the NICU can be divided into three parts: nursing prevention, department cooperation, and infection early warning.

Nursing prevention: 1) Equipment management needs to be strengthened, especially the disinfection management of incubators must be handled by the “Technical Specifications for Disinfection in Medical Institutions” (Jing et al., 2020) to ensure aseptic operation. The water tank is disinfected in time, and communication with the inspection department is often carried out, as well as the incubator must meet the standard before it can be used. 2) For those infected, they should be isolated. The materials, equipment, instruments, sinks, and bathtubs used by the child should be checked and prohibited from being mixed with others. When operating, the movements need to be gentle to reduce bleeding. If necessary, special area care should be taken to reduce aerosolization, and local bleeding should be disinfected with a 3% hydrogen peroxide solution. 3) If the newborn is abnormally crying, frowning, or turning over, it is necessary to actively find the cause. For example, if the umbilical area is infected, the natural shedding method can be used to minimize human intervention.

Department cooperation: 1) The combination of medical care and nursing should be insisted, and the internal medicine nurse and the head nurse should jointly evaluate the infection risk, which includes risk factors such as neonatal hypoglycemia, low immunity, and respiratory symptoms. 2) The infection prevention and control system in the NICU should be further improved, which includes rational use of antibiotics, disinfection equipment and supply management. Common diseases such as umbilical cord infections and urinary tract infections need to be paid close attention to, and preventive measures should be combined to prevent and treat a variety of bacterial infections.

Infection warning: 1) The assessment of risk factors such as the aforementioned low immunity. 2) The signs of infection such as fever, perforation, etc. For pain symptoms, the prenatal pain scale, the neonatal pain score, and the neonatal facial coding system should be used flexibly for daily assessment (Choi et al., 2017).

## 4 Evaluation of wireless physiological sensor network in ward

**FIGURE 5:**
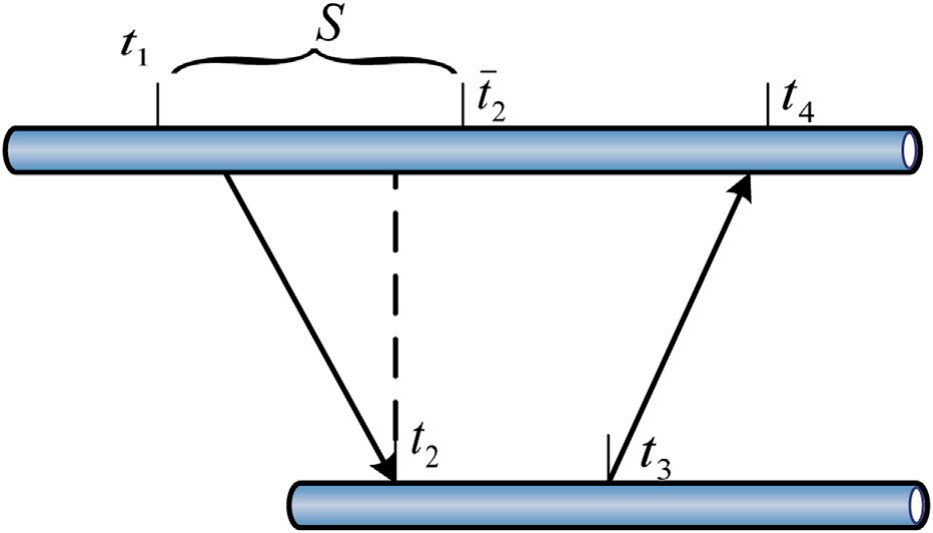
Schematic diagram of the TPSN algorithm.

Since the monitoring and early warning work in the Neonatal Intensive Care Unit are all carried out by a wireless network, it is necessary to analyze the wireless physiological sensor network. The wireless physiological sensor network is based on the wireless sensor network. The sensor collects physiological information through network nodes and transmits it to the ward monitoring terminal so that medical staff can monitor the condition of the newborn at any time and achieve early detection and early treatment. However, due to the uncertainty of the network transmission delay, the arrival time of the data packet cannot be used as the time of the sensor node data. Therefore, a clock synchronized with the central node must be maintained centrally in the sensor nodes, which is also called time synchronization. Timing-sync Protocol for Sensor Networks (TPSN) protocol is also called the TPSN algorithm. Compared with other time synchronization algorithms, it is more suitable for wireless physiological sensors in terms of synchronization error and synchronization accuracy. The TPSN algorithm adopts the mode of time synchronization, which is similar to the traditional network time protocol. Its purpose is to provide node time synchronization throughout the network. The algorithm has the advantage of high precision and is more suitable for the environment that requires high synchronization precision. The TPSN algorithm adopts a hierarchical network structure. First, all nodes are layered according to the hierarchical structure, and the time synchronization between all nodes and the root node is finally realized through the time synchronization between the lower node and its upper node. The schematic diagram of the TSPN algorithm is presented in Figure 5.

Node 1 belongs to the i−1 -level node and node 2 belongs to the i -level node; $t_{1}$ and represents the time measured by the local clock of node 2 at different times, and represents the time measured by the local clock of node 1 at different times; ε represents the time offset between two nodes, and represents the propagation delay of the message. Node 2 is the node to be synchronized, and node 1 is the reference node. Node 2 sends a synchronization request packet to node 1 at time $t_{1}$ , and the packet contains the level of node 2 and time $t_{1}$ . Node 1 receives the packet at a time $t_{2}$ , and At with me $t_{3}$ , a response packet is sent to node 2, and the packet contains the level of node 1 and the information of $t_{1}$ , and $t_{3}$ . The node receives the response at ta id $t_{4}=t_{3}+S-\varepsilon$ , so the following formulas can be derived:

$$S=\frac{\left(t_{2}-t_{1}\right)+\left(t_{4}-t_{3}\right)}{2}$$ (1)

$$\varepsilon =\frac{\left(t_{2}-t_{1}\right)-\left(t_{4}-t_{3}\right)}{2}$$ (2)

Node 2 uses Formula 1, 2 to estimate the round-trip delay S and time offset and adjusts its clock according to the values of and ε ( $t_{1}$ is the local clock of node 1, $t_{2}$ is the time of node 2), Finally, the purpose of synchronization with reference node 1 is achieved.

From the description of the TPSN algorithm, it can be known that: The error consists of transmission delay and time deviation. The transmission delay is the deterministic part, and the time deviation is the uncertainty part. If the clock frequency is not consistent, it would also cause time drift; therefore, the non-deterministic part consists of time offset and time drift. To reduce the synchronization error caused by the uncertain part and improve the synchronization accuracy, the least squares method is used to optimize the TPSN algorithm.

It is assumed that the propagation delay is and the time offset is ε . If the actual reception time is $t_{2}$ and the time corresponding to node 2 is ${\bar{t}}_{2}$ , let the formula be:

$${\bar{t}}_{2}=t_{1}+S$$ (3)

From Formula 1, the following formula can be obtained:

$${\bar{t}}_{2}=\frac{t_{1}+t_{2}+t_{4}-t_{3}}{2}$$ (4)

To find the time deviation and drift, the mathematical model is established:

$$t_{2}=\alpha {\bar{t}}_{2}+\varepsilon$$ (5)

In the formula, the corresponding time drift of the two nodes; ε is the time deviation. It is assumed that i is the i th synchronization, and Formula 5 can be arranged as:

$$t_{i2}=\alpha \frac{t_{i1}-t_{i3}+t_{i2}t_{i4}}{2}+\varepsilon ,i=1,2,\ldots ,n$$ (6)

There are two parameters in the formula, which can be solved by the least square method (Jing et al., 2020).

Let the formulas be:

$$y_{i}=t_{i2}$$ (7)

$$x_{i}=\frac{t_{i1}-t_{i3}+t_{i2}-t_{i4}}{2}$$ (8)

The following formula can be obtained:

$$y_{i}=\alpha x_{i}+\varepsilon$$ (9)

The least squares method is used to find the estimates and of the unknown parameters and ε , and is made the best fit of all.

The deviation of the estimated value from the actual value is:

$$\delta_{i}=y_{i}-{\vec{y}}_{i}=y_{i}-\vec{\alpha }x_{i}-\vec{\varepsilon }$$ (10)

The sum of squared deviations is used to quantitatively describe how well fits all scatter points $\left(x_{i},y_{i}\right)\left(i=1,2,\cdots ,n\right)$ . Q changes with and y . The best fit of a line with scatter points is to find any of the minimum of Q , which is also to make $Q\left(\vec{\alpha },\vec{\varepsilon }\right)=\min Q\left(\alpha ,\varepsilon \right)$ . By using the extreme value method in calculus, the following formula can be obtained by calculating $\vec{\alpha }$ and $\vec{\varepsilon }$ (Song et al., 2018; Maclean et al., 2021):

$$\left\{\begin{array}{l} \frac{\partial Q}{\partial \vec{\varepsilon }}=-2\sum_{i=1}^{n}\left[y_{i}-\left(\vec{\alpha }x_{i}+\vec{\varepsilon }\right)\right]=0\\ \frac{\partial Q}{\partial \vec{\alpha }}=-2\sum_{i=1}^{n}\left[y_{i}-\left(\vec{\alpha }x_{i}+\vec{\varepsilon }\right)\right]x_{i}=0 \end{array}\right.$$ (11)

After simplification, the following formulas can be obtained:

$$n\vec{\varepsilon }+\alpha \sum_{i=1}^{n}x_{i}=\sum_{i=1}^{n}y_{i}$$ (12)

$$\bar{\varepsilon }\sum_{i=1}^{n}x_{i}+\vec{\alpha }\sum_{i=1}^{n}x_{i}^{2}=\sum_{i=1}^{n}x_{i}y_{i}$$ (13)

The estimated values for solving are:

$$\alpha =\vec{\alpha }=\frac{\sum_{i=1}^{n}\left(x_{i}-x\right)\left(y_{i}-y\right)}{\sum_{i=1}^{n}{\left(x_{i}-x\right)}^{2}}$$ (14)

$$\varepsilon =\vec{\varepsilon }=y-\alpha x$$ (15)

Node 2 can adjust its local time according to the time offset and time drift to achieve time synchronization. In this way, the time deviation can be kept unchanged for a long time to achieve the purpose of reducing energy consumption, and it can also ensure that the transmission of the wireless physiological sensor network and the collection of physiological data can be carried out synchronously.

## 5 Evaluation of experimental results under the new method of infection prevention and control in neonatal intensive care unit

In this study, physiological sensors are utilized for infection control and early detection in the neonatal intensive care unit, leading to the development of a novel approach to ward infection prevention and control. 300 people are first asked about their opinions of the new procedure to understand the application effect of it. There are 100 medical professionals, nurses, and family members among the 300 persons. The survey results are shown in Table 1.

**TABLE 1:** Satisfaction status of medical staff and family members with new prevention and treatment methods.

Satisfaction level	Doctor		Nurse		Family	
	Number of people	Proportion (%)	Number of people	Proportion (%)	Number of people	Proportion (%)
dissatisfied	7	2.3	3	1	9	3
satisfy	26	8.7	35	11.7	27	9
Very satisfied	67	22.3	62	20.7	64	21.3

From the data in Table 1, it can be seen that whether it is medical staff or family members, the number of satisfied and very satisfied accounts for a large proportion. The number of people in the three types of people who are very satisfied exceeds 20% of the total number of people, and the number of people who are dissatisfied does not exceed 5%. Comparing the proportion of the number of people, it can be seen that the satisfaction level of medical staff and family members with the new method is still relatively high, which shows that the implementation of the new method is recognized by everyone.

**FIGURE 6:**
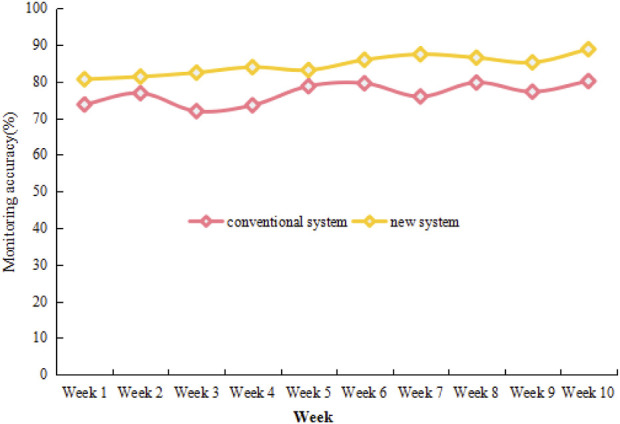
Monitoring accuracy rates under the conventional system and the new system in 10 weeks.

In the method part, a new ward monitoring system is constructed using physiological sensors, and the ultimate purpose is to monitor the physiological data of newborns. To verify whether the system can perform accurate monitoring, the monitoring accuracy of the system is analyzed by taking the newborns in the Intensive Care Unit of a hospital as the monitoring object, and compared with the conventional system monitoring. The specific time is 10 weeks, as shown in Figure 6.

It can be seen that the monitoring accuracy rate under the conventional system is maintained between 70% and 80% in 10 weeks, and the fluctuation trend of the accuracy rate each week is more obvious. In contrast, the monitoring accuracy rate under the new system exceeds 80% in the first week, and the accuracy rate for the next 9 weeks also remains above 80%, as well as the fluctuation trend is small. In the 10th week, the monitoring accuracy rate even exceeds 88%. According to the trend of the line chart, it can be concluded that the new system has a good performance in monitoring accuracy.

**FIGURE 7:**
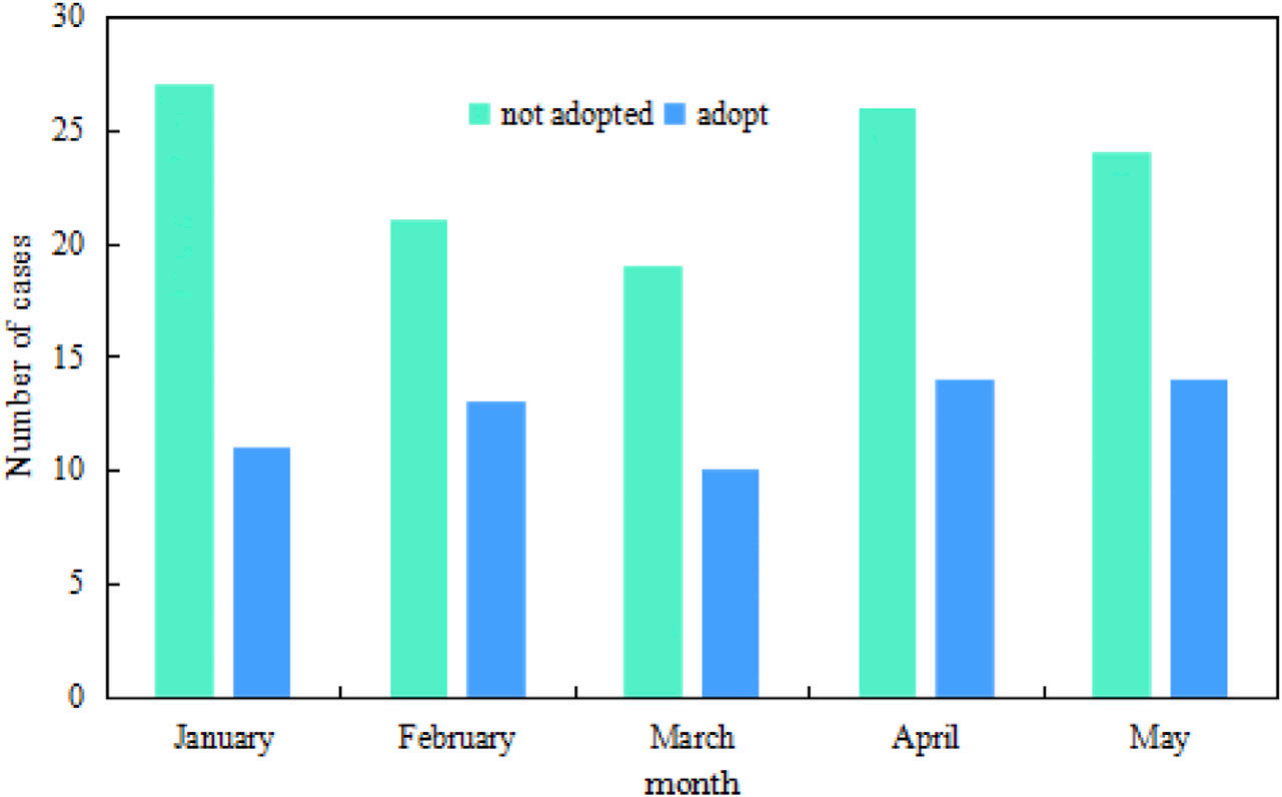
Changes in the number of neonatal morbidities from January to May.

**FIGURE 8:**
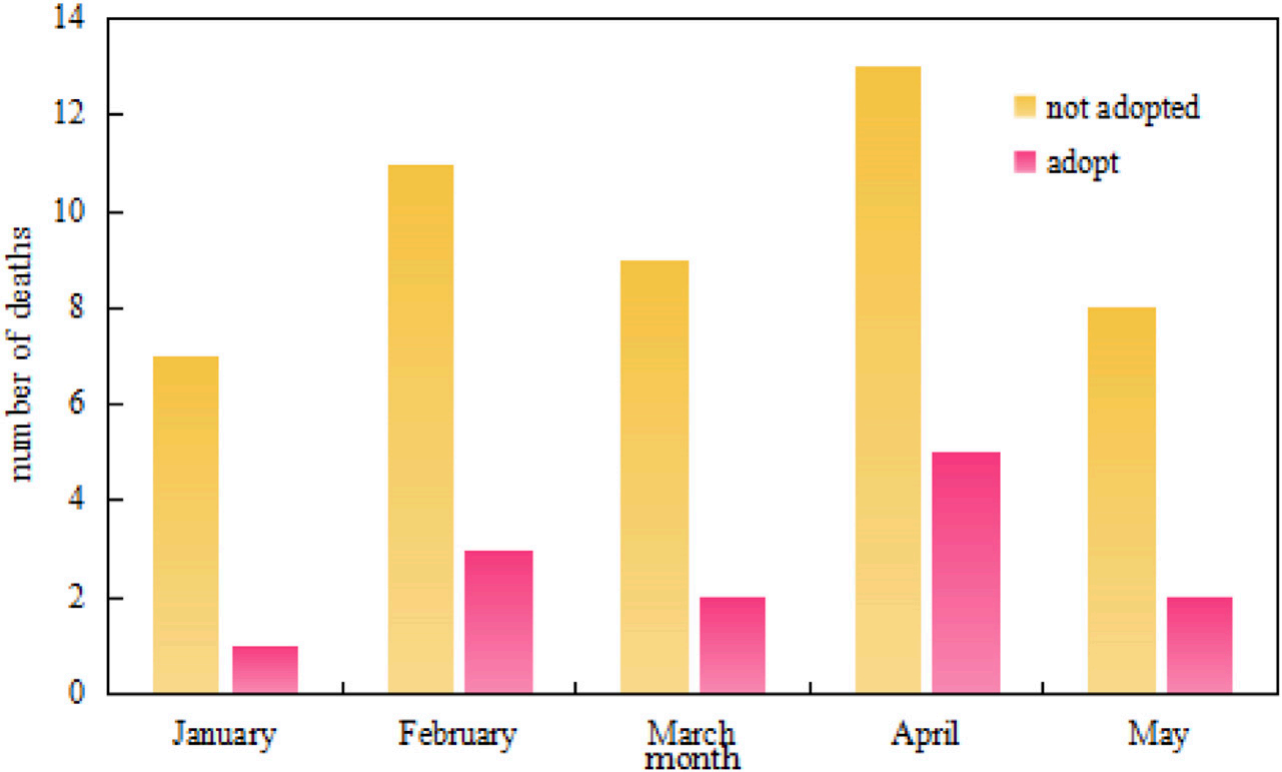
Changes in the number of neonatal deaths from January to May.

The infection prevention method in NICU proposed in this paper includes monitoring and early warning systems and protective measures, which can achieve better results by taking both measures. To understand the practical effect of the new method, the number of morbidity and deaths among 1000 neonates in a hospital’s Neonatal Intensive Care Unit with or without the new method is investigated. The specific time is from January to May, and the survey results are shown in Figure 7 and Figure 8.

In the bar chart, Figure 7 shows the number of neonatal morbidities from January to May, and Figure 8 shows the number of neonatal deaths from January to May. It can be seen that the number of cases and deaths varies from month to month. When no new control method is adopted, the peak number of cases exceeds 25 and the death toll exceeds 12. After adopting the new control method, the number of cases drops to less than 15 at the peak, and the death toll drops to less than 6 at the peak. Comparing the number of people at 5 months, it can be concluded that the use of new prevention methods can reduce the number of morbidity and deaths in the NICU to a certain extent.

**FIGURE 9:**
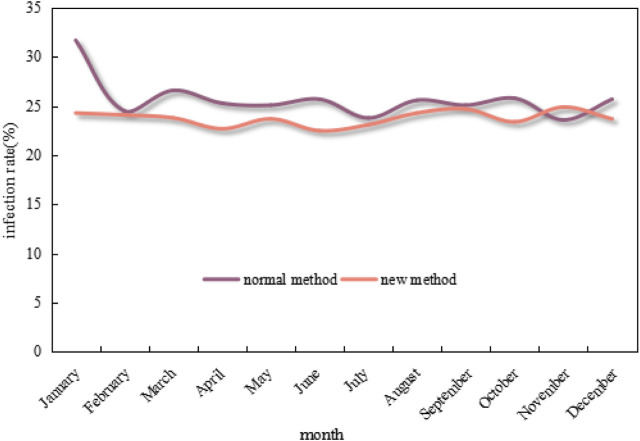
Changes in neonatal ward infection rates under the conventional method and the new method in 12 months.

Figure 9 shows the 12-month changes in neonatal ward infection rates under the conventional approach and the new approach.

As can be seen from the graph in Figure 9, the infection rate of neonatal wards in 12 months under the conventional method mostly exceeds 25%, which even exceeds 30% in January. In contrast, the infection rate under the new method is much lower and mostly maintains below 25%, as well as drops to below 22% at the lowest point. Overall, the infection rate under the new method is 7.39% lower than that of the conventional method.

## 6 Conclusion

Family members increasingly need assistance in caring for critically unwell newborns as medical and living standards advance. Neonatal Intensive Care Units are becoming commonplace in hospitals, which not only makes it easier to treat babies but also poses a concealed risk of ward infection. An effective intelligent monitoring system is required to have a thorough and precise understanding of the newborn’s physiological data. At present, the monitoring system equipment in the hospital is old and slow, which brings difficulties to the monitoring work. Coupled with the lack of timely updating of preventive measures, the infection rate in the ward would increase. The application of physiological sensors to infection prevention and early warning in the Neonatal Intensive Care Unit is a breakthrough, which is of great significance to the prevention and control of ward infection.

## Data Availability

The original contributions presented in the study are included in the article/Supplementary material, further inquiries can be directed to the corresponding authors.
